# Improving the emotional wellbeing of university students through culturally adapted cognitive and dialectical behavioral group interventions: protocol for two parallel feasibility and effectiveness studies

**DOI:** 10.1093/tbm/ibag010

**Published:** 2026-03-24

**Authors:** Ayse Akan, Nazli Hilal Korkut

**Affiliations:** Translational Clinical Psychology Lab, Department of Psychology, Boğaziçi University, Istanbul, Turkey; Translational Clinical Psychology Lab, Boğaziçi University, Istanbul, Turkey

**Keywords:** CBT, cultural adaptation, DBT, group therapy, mental health, university students

## Abstract

**Background:**

University is a period of increased vulnerability to mental health challenges, with anxiety and difficulties in emotion regulation among the most prevalent. Culturally adapted Cognitive Behavioral Therapy (CBT) and Dialectical Behavior Therapy (DBT) group interventions can support student well-being, yet scalable options remain limited. To address this gap, group CBT for anxiety management and group DBT for emotion regulation were culturally adapted for university students in Türkiye. Adaptations involved revising content and language, removing culturally mismatched elements, and incorporating culturally relevant and age-appropriate material.

**Purpose:**

This project aims to assess the feasibility and effectiveness of delivering culturally adapted group CBT for anxiety management and group DBT for emotion regulation.

**Methods:**

The project includes three steps: (i) a comprehensive systematic review of group CBT and DBT interventions worldwide; (ii) translation, adaptation, localization, and development of 8-session group interventions; and (iii) implementation, data collection, and analysis. Participants are allocated to either the CBT group for anxiety management or the DBT group for emotion regulation.

**Results:**

Standardized measures of anxiety or emotion regulation are administered at baseline, post-intervention, and six-week follow-up for both experimental and waitlist arms. Qualitative data are collected through semi-structured interviews conducted after intervention completion.

**Conclusions:**

In this project, the feasibility and effectiveness of culturally adapted CBT and DBT group interventions for university students in Türkiye will be evaluated. Findings will inform wider-scale application of evidence-based therapies in university counseling centres in Global South and guide the design of a future randomized controlled trial.

**Clinical Trials Information:**

NCT07096154 and NCT07096141 on clinicaltrials.gov.

## Graphical abstract

**Graphical Abstract:**
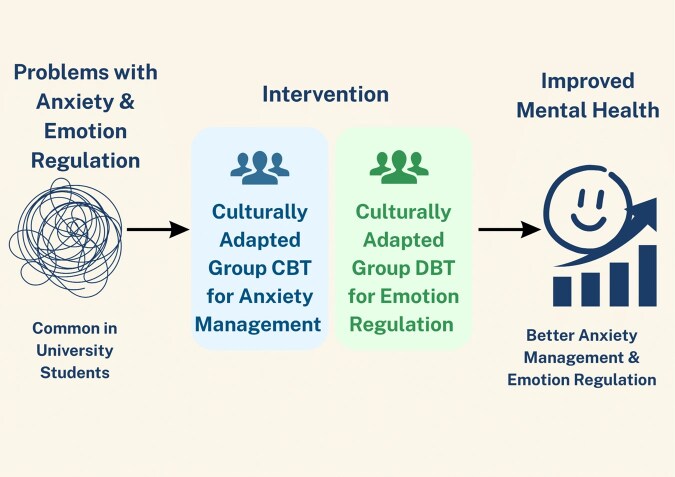

## Teaser

Group Cognitive Behavioral Therapy (CBT) for anxiety management and group Dialectical Behavior Therapy for emotion regulation interventions are culturally adapted to promote mental well-being of university students.

Implications

- **Practice:** University counseling centres can effectively address anxiety and emotion regulation challenges by integrating culturally adapted Cognitive Behavioral Therapy (CBT) and Dialectical behavior Therapy (DBT) group programs into their routine services.
- **Policy:** Higher education and public health authorities should consider supporting the integration of culturally adapted evidence-based interventions into university mental health services to make them more relevant and accessible.
- **Research:** More cultural adaptations of evidence-based therapies need to be investigated and trials across diverse universities should be conducted.

## Introduction

### Mental health of Turkish university students

University students’ psychological well-being is a prominent and growing public health issue worldwide [1]. The university period is a critical time for psychological, social and academic changes which may leave individuals more vulnerable to developing mental health problems [1]. It is suggested that almost one-third of students experience symptoms of common mental health disorders upon starting university [2]. In addition, students’ demand for university mental health support services has been increasing, which is above the rate of increase in the number of university students [3].

Research shows that university students in Türkiye are specifically at risk in terms of mental health in comparison to other countries. A systematic review covering countries such as China, Bangladesh, Spain, Russia, Korea, the US, and Japan reported a prevalence of 29% for anxiety, 37% for depression, and 23% for stress among university students, while in Türkiye, university students had much higher rates with 84.2% high stress, 36.2% high generalized anxiety symptoms, and 55% high depressive symptoms [4, 5]. Moderate and severe depressive symptoms of students in Türkiye have also been reported to be higher than the pooled international estimates [5]. Furthermore, national analyses conducted in Türkiye reveal that that 15–24 age group has the highest suicide rates which is consistent with WHO reports indicating that suicide is one of the top four causes of death among those aged 15–29 [6, 7].

### Access to mental health services in Türkiye

Despite the above-mentioned high rates of anxiety and emotion regulation difficulties, in Türkiye, accessible and free mental health services are usually restricted with 10-min psychiatric assessments, and psychological therapies are often a service people access paying out of pocket. In such circumstances, university mental health services become highly sought as some offer free or very low-cost psychological therapies. For this reason, evidence-based and cost-effective psychological therapies, such as group Cognitive Behavioral Therapy (CBT), and group Dialectical Behavior Therapy (DBT) informed interventions might constitute a solution.

### Group DBT informed and group CBT interventions

Research has shown that group based CBT and DBT can be effective intervention options that can promote mental well-being of university students [8, 9]. CBT has a significant evidence base established for treatment for anxiety disorders [10], and for this reason, recommended as first line of psychotherapy treatment by international health organizations such as National Institute for Health and Care Excellence [11, 12]. Research conducted internationally indicated the effectiveness of group CBT for anxiety management [13–15].

Group DBT interventions have been used and shown beneficial for emotion dysregulation related mental health difficulties [16]. Research has indicated that group DBT programmes can be effective for emotion regulation for university students and/or the 18–25 age group [17–23]. Furthermore, group CBT and DBT interventions provide a structure, foster companionship, limit the number of sessions, and can be cost and time effective for both the clients and the practitioners/services.

### Need for cultural adaptations for psychological therapies

It is accepted among mental health practitioners that an intervention that is customized for specific needs and contexts are more likely to be more helpful [24]. Furthermore, it is argued that Western conceptualisations of theories and interventions may not align with other cultures [25]. An example of this is that in many Western countries, therapist-client communication in therapy is on a first-name basis, whereas in Türkiye, clients often use more formal and hierarchical addresses such as “Ms/Mr/Dr” or “*Hocam*” (As an honorific title, *hoca* means “master” and is commonly used for teachers, professors, religious leaders, and in general, for wise people. It is also used as a slang word between friends.). This may indicate that participants in Türkiye may expect a more directive and mentorship-based intervention, at least at the beginning.

While following a very rigid fidelity to model approach in psychological interventions can provide structure and accountability, such rigidity also presents the risk of becoming an ineffective one-size-fits all method [24]. Various studies have suggested that culturally adapted interventions can promote positive mental health outcomes in different populations [26]. Cultural adaptation is not only about translation, it also necessitates localizing the structure and the delivery of the intervention tailored for the specific cultural context [27]. Psychological intervention methods developed in Western contexts may be less effective [24] not effective at all, and even harmful in different cultural contexts if values, norms and relationship dynamics are not considered.

However, despite seemingly desirable outcomes most of the group CBT and DBT interventions as explained above (see “Group DBT informed and group CBT interventions” section), these studies are predominantly conducted in the Global North, with minimal studies addressing adapting these therapy programmes for Turkish university students’ needs.

Although there are some studies investigating the effectiveness of CBT in reducing anxiety [28–32] and DBT in improving emotion regulation [33, 34] in Türkiye, feasibility studies in Turkish university students are scarce and little research has examined their practical implementation, engagement, and sustainability in real-world university settings, or analyzed clinical significance of the change. Furthermore, while some studies claim to have culturally adapted their interventions, they do not fully explain the localization process, making it difficult to assess the appropriateness and transferability of their methods [32–34]. Lastly, the unavailability of intervention manuals and protocols hinders replication and broader implementation.

To address this gap, it is essential for culturally adapted CBT and DBT group interventions to be created for Turkish university students and their effectiveness to be examined. We also hope that this study may serve as an example to other Global South countries with similar settings.

### Aims

This project aims to investigate the effectiveness of CBT based group anxiety and DBT-informed group emotion regulation interventions tailored for Turkish university students. The project also explores the feasibility of delivering these interventions in a group format within a university setting in Türkiye, and more specifically aims to: (i) test the feasibility and effectiveness of an adapted group CBT programme for anxiety management for Turkish university students; (ii) test the feasibility and effectiveness of an adapted group DBT-informed programme for emotion regulation for Turkish university students; (iii) investigate the thoughts, views, and experiences of Turkish university students who attended an adapted group CBT anxiety management intervention; (iv) investigate the thoughts, views, and experiences of Turkish university students who attended an adapted group DBT emotion regulation intervention.

Both interventions were systematically adapted to the cultural norms, lived experiences, and therapeutic needs of Turkish university students through a structured and progressive process. A detailed description of these adaptation procedures and concrete examples can be found in the Methods section (See “Second Stage—Cultural Adaptation of the Interventions” section).

## Method

### Project design

The project consists of three stages.

#### First stage—literature reviews

The first stage involves a comprehensive systematic review of group CBT and DBT interventions worldwide. Systematic reviews on the effectiveness of Cognitive Behavioral Therapy (CBT) based anxiety management groups and Dialectical Behavior Therapy (DBT) informed emotion regulation groups for university students were conducted to inform the intervention arm (third stage) of the study. Systematic review protocols are registered with Prospero with the registration numbers CRD42024590456 (CBT) and CRD42024575936 (DBT). Preliminary results indicate overall effectiveness for both interventions.

#### Second stage—cultural adaptation of the interventions

##### The team

Translation, adaptation, localization of an 8-session group CBT based anxiety management and DBT-informed emotion regulation interventions are led by a Turkish-speaking Clinical Psychologist and Assistant Professor (first author), and her team of Turkish trainee clinical psychologists, and trainee psychological counselors. Additionally, input was sought from a US trained and licensed Turkish Psychologist who has experience in university student mental health in the US and Türkiye.

The first author received her clinical psychology training in the UK, worked in the NHS with young people and young adults with anxiety and emotion regulation difficulties, and has supervisory experience in the UK and Türkiye. The team of trainee clinical psychologists and trainee psychological counselors are all educated and trained in Türkiye, thus, the entire team has direct familiarity with the cultural and educational context.

##### The framework

Barrero and Castro’s Heuristic Framework for its comprehensive, multidimensional and systematic structure was followed [35]. Consequently, the adaptation process consisted of the four stages: information gathering, preliminary adaptation design, preliminary adaptation tests and adaptation refinement. For the initial information gathering, comprehensive systematic reviews were conducted for group CBT and DBT interventions. Additionally, the needs and cultural context of the university population in Türkiye were assessed by the team through additional literature reviews, clinical observations, and expert opinions. Based on these findings, a preliminary adaptation design was created for both interventions by culturally adapting the language, examples, session content, and materials used in the intervention. As the cultural adaptation process do not end in this stage, further adjustments will be then made based on feedback obtained from participants and group facilitators during and after the feasibility trials, which is also recommended by Barrera Jr. & Castro [35]. The initial adaptation process for each group intervention is being further elaborated below.

##### Adaptation process for group CBT intervention for anxiety management

Group CBT for Anxiety Management is an 8-session intervention. Throughout the sessions, participants will be introduced to how CBT addresses anxiety and examine the connections between thoughts, emotions, physical responses, and behaviors. They will investigate the physiological effects of anxiety and practice relaxation techniques (e.g. breathing exercises, grounding techniques, progressive muscle relaxation). Participants will also learn to recognize their automatic thoughts and unhelpful thought patterns and will be equipped with effective strategies to challenge and reframe them. Additionally, they will strengthen their anxiety management skills through exposure to fear hierarchies and behavioral experiments. Finally, participants will be invited to decide the content of the seventh session to make it even more relatable to them.

The materials for CBT intervention has been selected by the first author using Beck’s and Padesky’s books [36, 37], choosing the components of CBT most relevant to the needs of our target population, rather than adapting the entirety of the resources. In this selection process, criteria such as feasibility of being able to be delivered in an 8-session group format, developmental characteristics of the population and suitability for the age group, and the applicability and the skill-focused structure of the content were considered. For example, techniques that require longer and more individualized therapeutic process or are difficult to implement in a group setting were not included. Examples that reflect the developmental characteristics of the population (e.g. identity formation, academic life, peer and romantic relationships, uncertainties about the future) were included in the content; examples that were developmentally less relevant to the university student population (e.g. parenting) were excluded so that the content would directly reflect and represent participants’ own experiences. These materials have been then translated into Turkish by the team of bilingual trainee clinical psychologists and trainee psychological counselors. The translated materials were then heavily edited and adapted to accommodate Turkish university students’ needs by the first author. Afterwards, the materials were checked by the rest of the team and changes were made based on the feedback the team provided.

The original framework of the intervention was retained, however, the first author created new examples that align with common experiences of university students in Türkiye such as bilingual education, academic worries, future and career related anxiety, relational and interpersonal problems, family conflicts and concerns related to developing autonomy from parents and family. Content and examples specific to this particular university, such as navigating university clubs, were also added. Additionally, CBT materials are prepared in a way that will allow group facilitators to bring their own examples. Considering that therapist self-disclosure often makes clients feel supported and validated, this format is intended to serve as a model for participants, encourage them to share their own experiences, reduce potential embarrassment or negative feelings that may arise in a group setting. The dynamic structure of the materials will facilitate better understanding and internalization of the content and provide data that will guide further refinement of the adaptation. For example, a facilitator might share how anxious they felt on a specific topic, and how they navigated this using CBT strategies. They will then encourage the participants to share their own examples. The impromptu examples brought by the facilitators then will be discussed in supervision to evaluate their suitability. Helpful examples that are consistent with the intervention framework may be incorporated into the ongoing adaptation of the protocol.

##### Adaptation process for group DBT intervention for emotion regulation

Culturally adapted group DBT-informed intervention for emotion regulation is designed to include 8 sessions, comprising the modules of mindfulness and emotion regulation [38]. Rather than adapting standard DBT for adults, session materials were derived from DBT for Adolescents, keeping in mind the age and this population’s dependency on their families. Mindfulness and emotion regulation modules of DBT [38] were selected by the first author because they are directly related to the focus of the intervention and are expected to be most effective in elevating the emotion regulation difficulties experienced by the university students. The criteria for this selection process included suitability for the 8-session group format, developmental characteristics, and age group appropriateness of the population, as well as the applicability of the content and its focus on skill building. These materials have been then translated into Turkish by the team of bilingual trainee clinical psychologists and trainee psychological counselors. The translated materials were then heavily edited and adapted to accommodate Turkish university students’ needs by the first author. Afterwards, the materials were checked by the rest of the team and changes were made based on the feedback the team provided. Furthermore, although the original manual includes parent-focused content, the first author heavily modified these sections, discarding any elements that could not be implemented within the group sessions.

Through the sessions, participants will work through the main problem areas that the DBT programme focuses on, familiarize with the three states of mind (emotion mind, reasonable mind, wise mind), practice mindfulness skills, get a better understanding of emotions and their functions, and work on improving emotion regulation skills through different exercises. To ensure cultural relevance, the content was systematically modified to include culturally relevant examples while maintaining the original framework of DBT. For example, Western political and popular culture references were replaced with Turkish ones. Furthermore, specific university-context elements were added including examples about campus-related situations, dormitory accommodation, interpersonal dynamics with faculty members, or dealing with academic workload. Everyday life examples were also modified by replacing culturally unfit elements with more relevant examples and scenarios to better reflect students’ and facilitators’ experiences (e.g. Turkish food instead of American food, using public transportation instead of driving, roommates/siblings instead of spouses). Additionally, to make the content more appropriate to the age group, elements related to video games, social media platforms (e.g. TikTok, Instagram) and online interpersonal behaviors (e.g. “ghosting,” “stalking,” “unfollowing”), which are highly relevant in lives of the young population today, were added. Also, based on the clinical anecdotes from the university counseling centre suggesting a significant number of neurodiverse students, selected sections were expanded to better reflect their experiences. Given the higher prevalence of sleep problems in this population, examples related to sleep were incorporated when addressing anxiety and emotion regulation. Additionally, relevant sections address sensory sensitivities in relation to emotional distress and anxiety. The material was also adapted to include other challenges neurodiverse individuals experience such as difficulty in planning, procrastination, sustained attention, time management, and social difficulty related anxiety and emotional regulation issues. Abbreviations, and acronyms were adapted to preserve both phonetic consistency and meaning in Turkish, ensuring that the content is engaging and impactful.

#### Third stage—implementation of culturally adapted interventions

The effectiveness and feasibility of both interventions will be investigated. This stage adopts a mixed-methods non-randomized pre-post design with a waitlist control and approximately 6 weeks follow-up. These feasibility studies are registered on clinicaltrials.gov with the identification numbers NCT07096154 and NCT07096141 for group CBT and group DBT interventions respectively.

### Participants

Up to 60 participants (experimental and control arms) for each study will be recruited from a university in Türkiye. We based our sample size on the study by Teare *et al.* [39], which recommended 60 participants per group for stable estimates of binary feasibility outcomes. We also aimed to maintain a minimum of 12 participants per group, as suggested by Julious [40], even in the worst-case scenario. Furthermore, it is a pragmatic decision considering the number of group facilitators needed to facilitate the groups and how many people we could include in each group.

### Inclusion and exclusion criteria

Any student enrolled in this particular university who is 18 years or older is eligible to attend the study. Serious and severe mental illness and high risk of self-harm/harm to others would be exclusion criteria as these individuals may require a more individual and tailored support and would be unlikely to benefit from a standalone group intervention. Scoring below the threshold of Beck Anxiety Inventory (BAI) for CBT study and Difficulties in Emotion Regulation Scale (DERS) for DBT study constitutes another exclusion criteria.

### Procedure

The language of the interventions, questionnaires, and the interviews are in Turkish. University’s psychological counseling centre is a stakeholder. An email invitation addressing the students and asking them to attend anxiety management or emotion regulation groups will be sent via the university’s psychological counseling centre. The volunteers who meet the clinical threshold for their respective groups will be invited to the groups on a first come first serve basis and receive either a group CBT intervention for anxiety-management or a group DBT-informed psychotherapy intervention for emotion regulation, based on their preference. If there are any volunteers who cannot be invited to the initial groups due to capacity, they will be placed on a waitlist and will be informed of this. All individuals who applied to join the groups will have eventual access to the intervention.

The interventions will last eight sessions and will be held on-site at campus in the university’s psychological counseling centre’s group rooms. The participants will not be allowed to attend the anxiety management and emotion regulation groups at the same time. The therapy groups will be facilitated by trainee or qualified clinical psychologists and/or trainee or qualified psychological counselors supervised by the first author. Data collection will be conducted online and in-session. For in-session quantitative data collection, separate research assistants will be employed. Further information on data-collection is provided below under “Data collection” section.

Participants will be asked to complete a batch of questionnaires at baseline, during and after the intervention, and after approximately 6 weeks follow-up. Furthermore, following the completion of the groups, qualitative data will be collected to explore the participants’ experiences and views on the group intervention they received. Although the study does not include an active control group, the waitlist controls will provide a reference condition that allows for comparison with the intervention group and enable a more accurate evaluation of the results. Tables 1 and 2 present full schedules of the assessment plans.

**Table 1:** Assessment Plan for the CBT group.

	Baseline assessment (t0)	Weekly assessment	Post-intervention assessment (t1)	Follow-up assessment (t2)
Socio-demographic Form	X			
Depression Anxiety and Stress Scale (DASS-42)	X		X	X
Beck Anxiety Inventory (BAI)	X		X	X
Generalized Anxiety Disorder-7 (GAD-7)	X		X	X
Patient Health Questionnaire-9 (PHQ-9)	X		X	X
Outcome Rating Scale (ORS)		X		
Group Session Rating Scale (GSRS)		X		
Client Feedback Form			X	

*Note.* ORS will be completed at the beginning of each session. GSRS will be completed at the end of each session.

**Table 2:** Assessment plan for the DBT-informed group.

	Baseline assessment (t0)	Weekly assessment	Post-intervention assessment (t1)	Follow-up assessment (t2)
Socio-demographic Form	X			
Borderline Evaluation of Severity Over Time (BEST)	X		X	X
Difficulties in Emotion Regulation Scale (DERS)	X		X	X
Depression Anxiety and Stress Scale (DASS-42)	X		X	X
Generalized Anxiety Disorder-7 (GAD-7)	X		X	X
Patient Health Questionnaire-9 (PHQ-9)	X		X	X
Outcome Rating Scale (ORS)		X		
Group Session Rating Scale (GSRS)		X		
Client Feedback Form			X	

*Note.* ORS will be completed at the beginning of each session. GSRS will be completed at the end of each session.

### Data collection

As this is a mixed-methods study, standardized measures and qualitative interviews will be used for data collection.

The measures used in this project are self-report instruments. Digitally completed baseline questionnaires will be filled out independently by the participants through Qualtrics [41].

Questionnaires administered at the beginning and the end of each session after the completion of the intervention will be filled out on paper and will be administered by research assistants, who will be either undergraduate or postgraduate students in psychology or psychological counseling programs. The research assistants will receive a training session led by the first author on procedures, ethics, and supporting participants, as well as ongoing supervision.

All measures, except for ORS and GSRS, will initially be administered online, so no additional data collection framework is required. Information on qualitative interviews is detailed under “Qualitative interviews” section, and data protection is discussed under “Data protection, confidentiality, and safety” section.

The questionnaires for assessment are specifically selected, considering their high validity and reliability which have been demonstrated in studies conducted with university samples. For anxiety management groups, Depression Anxiety and Stress Scale (DASS-42; adapted to Turkish by Bilgel & Bayram [42]), Beck Anxiety Inventory (BAI; adapted to Turkish by Ulusoy *et al.* [43]), Generalized Anxiety Disorder-7 (GAD7; adapted by Konkan *et al.* [44]), Patient Health Questionnaire (PHQ9; adapted by Sari *et al.* [45]) will be asked to be completed once before and once after the intervention, and approximately 6 weeks after the completion of the intervention (follow-up).

For emotion regulation group, Borderline Evaluation of Severity Over Times (BEST; adapted to Turkish by Akın [46]), Difficulties in Emotion Regulation Scale (DERS; adapted to Turkish by Rugancı and Gençöz [47]); Depression Anxiety and Stress Scale (DASS-42; adapted to Turkish by Bilgel and Bayram [42]), Generalized Anxiety Disorder-7 (GAD7; adapted by Konkan *et al.* [44]), Patient Health Questionnaire (PHQ9; adapted by Sari *et al.* [45]) will be asked to be completed once before and once after the intervention, and approximately 6 weeks after the completion of the intervention (follow-up).

For both groups, the Outcome Rating Scale (ORS; created by Miller *et al.* [48] and translated by the project team with the permission of the creator) will be asked to be completed at the beginning of each session while Group Session Rating Scale (GSRS; created by Quirk *et al.* [49] and translated by the project team with the permission of the creator) will be asked to be completed at the end of each session. Additionally, a socio-demographic form will be asked to be completed before the intervention, and a participant feedback form put together by the project team at the end of the intervention.

#### Depression anxiety and stress scale

DASS-42 was developed by Lovibond and Lovibond [50] to measure depression, anxiety and stress and was adapted to Turkish by Bilgel and Bayram [42]. The 42-item scale demonstrated good validity and reliability for university students.

#### Beck anxiety inventory

BAI is a 21-item tool that is developed to measure the severity of anxiety [51]. The Turkish adaptation of BAI has good psychometric properties [43]. It is a valid and reliable measurement tool to assess anxiety in a university sample.

#### Generalized anxiety disorder-7

GAD-7 is a brief questionnaire developed by Spitzer *et al.* [52] to assess generalized anxiety symptoms. The Turkish adaptation of GAD-7 demonstrated high validity and reliability [44].

#### Patient health questionnaire-9

PHQ-9 is a widely used tool to measure the severity of depressive symptoms. The 9-item self-report measure was developed by Kroenke *et al.* [53] and adapted to Turkish by Sari *et al.* [45]. The Turkish PHQ-9 has been found reliable, making it suitable for evaluation of depression.

#### Borderline evaluation of severity over time

BEST is a self-report measure, designed by Pfohl *et al.* [54] to assess the severity of borderline personality disorder symptoms over time. The adaptation of the measure was carried out by Akın [46], it demonstrated high validity and reliability.

#### Difficulties in emotion regulation scale

DERS is a 36-item measure developed by Gratz & Roemer [55] to assess emotion regulation difficulties. It was adapted to Turkish by Rugancı & Gençöz [47], demonstrating good psychometric properties.

#### Outcome rating scale

The ORS was developed by Miller *et al.* [48] as a brief assessment tool to measure therapeutic progress. The scale is translated by the project team with the permission of the creator.

#### Group session rating scale

The GSRS was developed by Quirk *et al.* [49] to measure the group therapy process from the participants’ perspectives. The scale is translated by the project team with the permission of the creator.

#### Socio-demographic form

The socio-demographic form is prepared by the project team. The self-report questionnaire is used to gather information about the demographic characteristics of the participants including age, gender and income level. Data about the academic background of the participants including major, GPA, and academic success will also be obtained through the questionnaire.

#### Client feedback form

The client feedback form is a 4 point Likert scale with 10 items and a free text box for comments, developed by the first author who was inspired from a feedback form used at University of Essex Doctorate in Clinical Psychology Programme. It is used to gather information about the participants’ experience with the group facilitator and the intervention. The participants will give feedback about their satisfaction level with the service they received, and the answers will be collected anonymously. The form includes questions about the therapeutic process, the structure of the sessions, the therapist’s approach, and whether the support was helpful.

#### Qualitative interviews

Participants who attend the groups, including those who dropped out, will be invited to attend an interview for in-depth qualitative feedback after the intervention, including reasons for dropout if appropriate. Qualitative data will be collected from 6 to 20 participants from anxiety management and 6 to 20 participants from emotion regulation groups, where they will be invited to share their experiences and thoughts about the difficulties and the group they attended. Participation in the interviews is entirely voluntary, and potential participants are not to be rejected from the studies if they do not wish to attend an interview. The interviews will be recorded using either a voice recording device or a password protected computer with the written permission of the participants. The interviews will be transcribed manually by the trainees who conducted them. The sample questions to be asked to the participants are: (i) Could you tell us a little about the process (your mental health difficulties) that led you to apply to this group? (ii) Were you satisfied with the group? (iii) Which parts of the group were useful to you? (iv) Which parts of the group need to change (what you didn’t like)? (v) How was your relationship with the therapist and assistants? (vi) How was your relationship with the other participants of the group? (vii) What was the most important thing you learned from the group? (viii) Are there any other things that stood out to you? (ix) Would you recommend this group to other students with similar problems? (x) Do you think this group should be an ongoing service provided by the university?

## Data analysis

### Investigating the feasibility and acceptability of recruitment and delivery

To evaluate the acceptability and feasibility of these conducted groups, completion rates, number of attended sessions, drop-out rates, perceived burden and inconvenience of attending groups, and reasons for non-completion, the client feedback form and in-depth qualitative interviews will be investigated and/or analyzed.

### Investigating effectiveness

Quantitative data on anxiety, depression, stress, emotion regulation difficulties, borderline symptoms, and general wellbeing collected via questionnaires in Tables 1 and 2 will be analyzed using descriptive and inferential statistics, and clinically significant change analysis [56] to see whether there was meaningful change in participants’ psychological difficulties and wellbeing following the completion of the group intervention. Reflexive Thematic Analysis conducted using qualitative data will also inform the effectiveness.

## Ethics and data protection

Ethical clearance has been obtained from Bogazici University Institutional Review Board in Social Sciences and Humanities (SBINAREK) with the date 28/06/2024 and the number 2024-38. Amendments were then accepted on 17/01/2025. During the consent process, participants will be informed about the purpose of the study, the institutions supporting the study, and the structure of the UniWELL project. Additionally, participants will be informed about the duration and content of the group intervention and that they will be required to complete several questionnaires at separate times before, during, and after the intervention. Participants will also be informed that attending the group is entirely voluntary, that they will not receive compensation, and that choosing not to participate will not affect any services they receive from the university. Information will be provided about confidentiality procedures, how data will be stored securely, how personal information will be removed and anonymized, and how the anonymized data may later be used in scientific publications or further research. The consent process will also explain potential risks, such as the possibility of emotional discomfort when answering questionnaires, and outline the support options and crisis procedures available if needed. Finally, participants will be given the research team’s contact information and the address of the university ethics board so they can ask questions or seek clarification before deciding whether to participate. Additionally, for ethical reasons, every eligible participant, including the ones on the waitlist, will be offered a group intervention that will target their problems.

### Data protection, confidentiality, and safety

Quantitative data that are collected online will be fully anonymized after the follow up step and will be kept as an excel file on a password protected secure drive. Only the researchers who do not hold an academic position at the university will have access to identifiable information.

Quantitative data collected by using pen and paper will be locked securely in a locked cabinet in the university’s psychological counseling centre or in a campus-based office. The paper-based data will be destroyed after the information is entered on excel securely. Qualitative interview recordings will be kept securely on a password-protected device until transcriptions of the records made. Following that, the voice recordings will be permanently deleted, and the transcriptions will be stored securely on a password-protected drive. The anonymized quantitative data will be kept securely on a password protected drive for 10 years and will be shared with peer-reviewers if requested, while the qualitative interview transcripts will also be kept securely on a password protected drive but they will not be shared with peer-reviewers (will be only open to the research team) until it is deleted after 10 years. These data may be used in secondary analysis projects by some of the research team.

Participant name or any identifiable information including contact information will not to be shared in any publication or any other form of presentation. Additionally, the first author, will never have access to the names of the students who attended the study to manage their dual role as a researcher and instructor at the same university. The only exception is a high-risk situation, where a participant develops high suicidality and self-harm behaviors, and the trainee psychologists or counselors would not be able to handle the situation. If that is the case, information of the participant may be shared with people or organizations that would ensure the participant’s safety. This could include facilitating access to a psychiatric clinic or hospital. The participants will also be asked to provide an emergency contact number to ensure the participant’s safety and wellbeing in case of an emergency. This exception is included in the consent form.

## Limitations

There are potential limitations to this study. The intervention and data collection will be conducted at a single university, this may affect the generalizability of the data to different contexts. The results and outcomes may reflect the developmental, cultural, and institutional characteristics of this group and therefore may not be directly extended to different cultural contexts, age groups, educational settings, or socioeconomic backgrounds. Second, the study has a relatively small sample size, which may also affect the robustness of the results. Finally, although the study has a waiting list control group, it does not include an active control group, which may limit the ability to attribute the results to the intervention only. Nevertheless, by addressing anxiety and emotion regulation difficulties in a culturally adapted group format, the findings will provide valuable information and contribute to the development of effective mental health interventions in university settings. Future research could examine adaptation and implementation processes in multi-cite trials with active control arm throughout Türkiye, also recruiting larger samples.

## Discussion

It is well documented that university students are more vulnerable to experiencing psychological difficulties which indicates the need for providing students with accessible and effective mental health support [57], with Turkish university students’ need being one of the highest due to its very high rates of anxiety, stress and depressive symptoms [4, 5, 58]. Although a number of evidence-based interventions have been shown to be effective in managing anxiety and emotion regulation difficulties, culturally adapted versions of these interventions have not been developed or evaluated within the Turkish university population. Culturally adapted group CBT and DBT interventions, being cost-effective, evidence-based, and applicable in a university setting, can play an important role in supporting the mental well-being of university students, particularly in reducing the difficulties they experience in anxiety management and emotion regulation. The present feasibility trial therefore represents one of the first structured attempts to culturally adapt group CBT for anxiety management and group DBT for emotion regulation interventions for university students in Türkiye.

By conducting this project, we will have the opportunity to offer Turkish university students a culturally adapted, evidence-based, low-cost treatment. Additionally, the intentional dynamic design of the project will allow further refinement of the adaptation based on feedback from both the participants and facilitators. By investigating the feasibility and the effectiveness, we hope to contribute to growing scientific evidence for culturally adapted cognitive behavior interventions and promote said interventions to other university psychological counseling centres in Türkiye, and possibly in other Global South countries. Finally, further research is planned to be conducted adopting a Randomized Clinical Trial method in collaboration with other universities following the completion of this project.

## Data Availability

The anonymized quantitative data will be kept securely on a password protected drive for 10 years and will be shared with peer-reviewers if requested. The data supporting this study’s findings will be available from the corresponding author upon reasonable request, starting immediately after publication. Data will be maintained and available for 10 years after the last data collection. On the other hand, anonymized qualitative interview transcripts will also be kept securely on a password protected drive but they will not be shared with peer-reviewers (will be only open to the research team) until it is deleted after 10 years. Some of the research team may use these data in secondary analysis projects.

## Welfare of animals

This article does not contain any studies with animals performed by any of the authors.

## Transparency statements

### Study registration

The first registration attempt was made during the recruitment process. However, the actual registration finalized after the study began. The parallel studies are registered on clinicaltrials.gov and be found at https://clinicaltrials.gov/study/NCT07096141 and https://clinicaltrials.gov/study/NCT07096154.

### Analytic plan pre-registration

The analysis plan was not formally pre-registered.

### Analytic code availability

There is not analytic code associated with this study.

### Materials availability

Questionnaires are publicly available. Interview questions are being made available in this protocol. Culturally adapted intervention protocols in Turkish will be made available in a book/booklet/pdf format.

### AI

Artificial intelligence was not used in the development of this manuscript.
